# Diverse strategies of O_2_ usage for preventing photo-oxidative damage under CO_2_ limitation during algal photosynthesis

**DOI:** 10.1038/srep41022

**Published:** 2017-01-20

**Authors:** Ginga Shimakawa, Yusuke Matsuda, Kensuke Nakajima, Masahiro Tamoi, Shigeru Shigeoka, Chikahiro Miyake

**Affiliations:** 1 Department of Biological and Environmental Science, Faculty of Agriculture, Graduate School of Agricultural Science, Kobe University, 1-1 Rokkodai, Nada, Kobe 657-8501, Japan; 2 Research Center for the Development of Intelligent Self-Organized Biomaterials, Research Center for Environmental Bioscience, Department of Bioscience, Kwansei-Gakuin University, 2-1 Gakuen, Sanda, Hyogo 669-1337, Japan; 3 Department of Advanced Bioscience, Faculty of Agriculture, Kinki University, 3327-204 Nakamachi, Nara 631-8505, Japan

## Abstract

Photosynthesis produces chemical energy from photon energy in the photosynthetic electron transport and assimilates CO_2_ using the chemical energy. Thus, CO_2_ limitation causes an accumulation of excess energy, resulting in reactive oxygen species (ROS) which can cause oxidative damage to cells. O_2_ can be used as an alternative energy sink when oxygenic phototrophs are exposed to high light. Here, we examined the responses to CO_2_ limitation and O_2_ dependency of two secondary algae, *Euglena gracilis* and *Phaeodactylum tricornutum*. In *E. gracilis*, approximately half of the relative electron transport rate (ETR) of CO_2_-saturated photosynthesis was maintained and was uncoupled from photosynthesis under CO_2_ limitation. The ETR showed biphasic dependencies on O_2_ at high and low O_2_ concentrations. Conversely, in *P. tricornutum*, most relative ETR decreased in parallel with the photosynthetic O_2_ evolution rate in response to CO_2_ limitation. Instead, non-photochemical quenching was strongly activated under CO_2_ limitation in *P. tricornutum*. The results indicate that these secondary algae adopt different strategies to acclimatize to CO_2_ limitation, and that both strategies differ from those utilized by cyanobacteria and green algae. We summarize the diversity of strategies for prevention of photo-oxidative damage under CO_2_ limitation in cyanobacterial and algal photosynthesis.

Air consists of 21% O_2_, the concentration of which increased during the evolution of oxygenic phototrophs, in particular the oceanic cyanobacteria, around 2.3 billion years ago^1^. Due to its electron configuration, O_2_ has a very high oxidizing potential and is the final electron acceptor in aerobic respiratory electron transport.

Oxygenic photosynthesis uses photon energy to produce sugar from CO_2_ and H_2_O, and releases O_2_ as a waste product. Two photosystems, PSI and PSII, play central roles in this process, which involves an electron transport system located on thylakoid membranes. The reaction centers, P700 and P680, are photo-oxidized via light-harvesting pigments such as chlorophyll (Chl). The oxidized P700 in PSI accepts electrons from PSII via plastoquinone, the cytochrome *b*_6_/*f* complex, and plastocyanin (or cytochrome *c*_6_). This electron transport is accompanied by the generation of a proton gradient across the membranes (ΔpH), allowing the production of ATP by ATP synthase^2^. At the acceptor side of PSI, NADP^+^ is reduced to NADPH by accepting electrons from P700 through ferredoxin and ferredoxin-NADP^+^ reductase. O_2_ is produced via the oxidation of H_2_O by oxidized P680 in the luminal side of PSII. Together, these reactions are termed ‘photosynthetic electron transport’, and are the source of chemical energy in the forms NADPH and ATP, which are used for CO_2_ assimilation in the Calvin-Benson cycle^3^.

The production and consumption of energy by photosynthetic electron transport and the Calvin-Benson cycle becomes unbalanced without sufficient CO_2_ (CO_2_-limited photosynthesis; Fig. 1). Excess photon energy causes the production of reactive oxygen species (ROS), which trigger oxidative damage to PSII and PSI, so-called photoinhibition^4^,^5^,^6^,^7^.

It is broadly accepted that O_2_ is essential, not only as the respiratory electron acceptor, but also as an electron sink for various reactions of photosynthesis: O_2_-dependent alternative electron flow (AEF)^8^, including the Mehler-ascorbate peroxidase (MAP) pathway^9^,^10^, singlet O_2_ production in PSII^5^, flavodiiron protein (FLV) reactions^11^,^12^,^13^, mitochondrial respiration^14^, and plastidial (or cyanobacterial) respiration^15^,^16^. Also, photorespiration can be explained as an O_2_-dependent AEF in the broad sense^17^,^18^,^19^. A large O_2_-dependent AEF that replaces photosynthesis can alleviate photoinhibition by dissipating excess energy to O_2_^11^,^12^,^13^,^17^,^18^,^19^,^20^,^21^,^22^. Recently, we showed that an O_2_-dependent AEF is essential for the oxidation of P700 under CO_2_ limitation to protect PSI against photo-oxidative damage in the cyanobacterium *Synechococcus* sp. PCC 7002 (S. 7002)^22^,^23^. That is, oxygenic phototrophs accessed O_2_ to prevent photo-oxidative damage derived from O_2_. However, both the magnitude and the molecular mechanisms of O_2_-dependent AEF vary across species in oxygenic phototrophs^12^,^13^,^18^,^22^,^23^.

There are alternative mechanisms, which do not depend on O_2_, that dissipate excess photon energy in oxygenic phototrophs. First, during Chl fluorescence measurements, non-photochemical quenching (NPQ) is observed as a decrease in maximum Chl fluorescence yields (F_m_ or F_m_′). Simply put, NPQ is a process of heat dissipation of photon energy around PSII. The molecular mechanisms of NPQ in various oxygenic phototrophs are diverse and include the xanthophyll cycle, light-harvesting complex II aggregation, and state transition, some of which are activated by ΔpH^24^,^25^. The degree of induced NPQ varies widely depending on growth and measurement conditions^24^,^25^. Second, the electron transport in the cytochrome *b*_6_/*f* complex has suppressed sensitivity to ΔpH^26^ or reduced plastoquinone pool^27^, which is expected to oxidize PSI to alleviate the production of ROS by PSI owing to P700 quenching. Finally, cyclic electron transport (CET) around PSI supports the formation of ΔpH to induce NPQ and down-regulate the electron transport in the cytochrome *b*_6_/*f* complex^28^. We note that CET is defined as an AEF but does not require O_2_. In prokaryotic and eukaryotic algae, CET around PSI is suggested to be driven in several pathways, including chloroplast NADPH dehydrogenase (NDH) 1 and 2, and an elusive ferredoxin-plastoquinone reductase^29^. Further, CET around PSII has been found in the green alga *Chlorella pyrenoidosa*^30^,^31^.

In this study, we measured responses to CO_2_ limitation of the cyanobacterium *Synechococcus elongatus* PCC 7942 (S. 7942) and two secondary algae, the Euglenoid *Euglena gracilis* and the pennate marine diatom *Phaeodactylum tricornutum*. We aimed to elucidate the diversity of mechanisms to utilize O_2_ as an alternative electron acceptor in photosynthetic electron transport to CO_2_ in cyanobacteria and algae. Cyanobacteria are known as the ancestors of chloroplasts, and have evolved into the chloroplasts of various photosynthetic eukaryotes via endosymbiosis. In contrast, the secondary algae are known to be the products of two endosymbiotic events and to have evolved from cyanobacteria along different lineages from that of land plants^32^. Chloroplasts of *E. gracilis* are possibly derived from a green plastid-containing organism and are surrounded by a triple, rather than a double, membrane as found in vascular plants and green algae^32^, which possess Chl *a* and *b* as light-harvesting pigments. However, the relative content of Chl *b* to Chl *a* in *E. gracilis* is less than that in vascular plants^33^. Conversely, *P. tricornutum* harbors chloroplasts that possibly originated from red algae, and are surrounded by a quadruple membrane^32^. The light-harvesting complex of *P. tricornutum* has fucoxanthin-Chl *a*/*c* binding proteins containing the carotenoids diadinoxanthin and diatoxanthin, which are involved in NPQ^34^.

## Results

### Responses of photosynthetic electron transport to CO_2_ limitation in S. 7942, *E. gracilis*, and *P. tricornutum*

We measured O_2_ and relative Chl fluorescence in S. 7942, *E. gracilis*, and *P. tricornutum* using an O_2_ electrode and a PAM fluorometer to evaluate the responses of photosynthetic electron transport. We estimated AEF activities in S. 7942, *E. gracilis*, and *P. tricornutum* using the relationship between photosynthetic O_2_ evolution rates and the relative electron transport rate (ETR) at PSII. Photosynthetic O_2_ evolution rate reflects the activity of photosynthesis (the Calvin-Benson cycle), whereas relative ETR is related to total electron transport activity, including AEF. Actually, we have found that the deletion of FLV2 and 4 (FLV2/4), which is the molecular mechanism of AEF under CO_2_ limitation, gives the proportional linear relationship between photosynthetic O_2_ evolution rates and relative ETR in the cyanobacterium *Synechocystis* sp. PCC 6803 (S. 6803) (Supplemental Fig. S1)^13^. These rates showed proportional linearity in CO_2_-saturated conditions in the two secondary algae, but not in S. 7942 (Supplemental Figs S2–S4), which suggests that electron transports at PSII were strongly coupled to photosynthesis in *E. gracilis* and *P. tricornutum* when sufficient CO_2_ was available. In S. 7942, relative ETR was already partially uncoupled from photosynthetic O_2_ evolution rate during CO_2_-saturated photosynthesis at supersaturated photon flux densities (Supplemental Fig. S2), indicating that cyanobacterial AEF functions in such situations^35^. We note that AEF can be reflected in the relative ETR only when the AEF has a high activity level comparable to photosynthesis.

**Figure 2:**
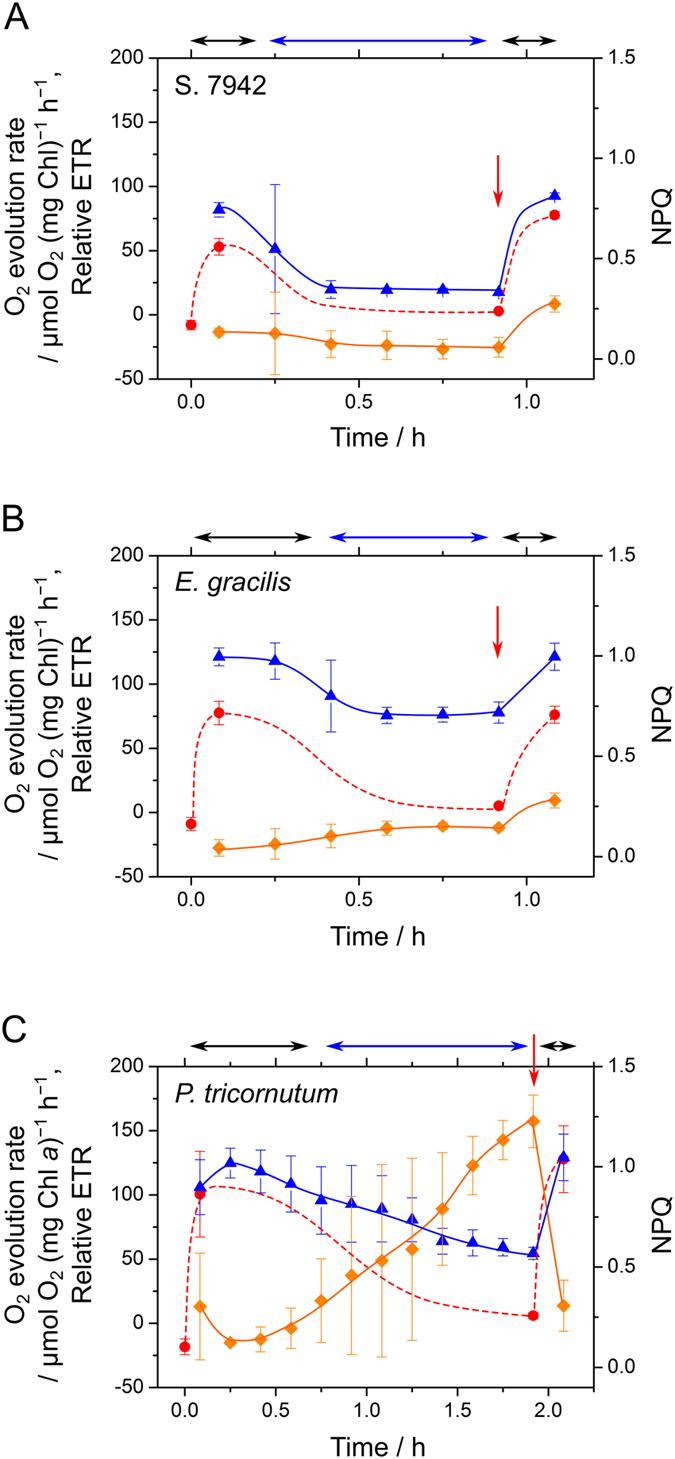
Responses of photosynthesis to CO_2_ limitation in S. 7942 (**A**), *Euglena gracilis* (**B**), and *Phaeodactylum tricornutum* (**C**). The graphs show the time course of O_2_ evolution rate (red circles), relative electron transport rate (ETR) (blue triangles), and non-photochemical quenching (NPQ) (orange diamonds) in the fresh media containing the cells (10 μg Chl *a* mL^−1^). Illumination with white actinic light (AL) (300 μmol photons m^−2^ s^−1^ for S. 7942 and *E. gracilis*; 400 μmol photons m^−2^ s^−1^ for *P. tricornutum*) began at 0. NaHCO_3_ (final concentration 10 mM) was added at the times indicated by red arrows. The double-headed black and blue arrows show CO_2_-saturated and CO_2_-limited photosynthesis, respectively.

Responses of algal photosynthesis to CO_2_ limitation were measured by following the method previously described^12^,^13^. The responses of photosynthetic parameters to CO_2_ limitation are shown in Fig. 2, and the original traces used to estimate these parameters are presented in Supplemental Figs S5–S7. The cyanobacterial and algal cells in fresh media were applied to an O_2_ electrode chamber without adding inorganic carbon sources, and then illuminated with white actinic light (AL). Illumination with AL stimulated photosynthesis, which was accompanied by an increase in O_2_ in the reaction medium (Supplemental Figs S5–S7). However, CO_2_ in the medium was gradually removed by algal photosynthesis, as the diffusion of CO_2_ from the atmosphere into the reaction medium was very slow, compared with the consumption by photosynthetic CO_2_ assimilation in the experimental system. O_2_ in the reaction medium began to decrease when CO_2_ was depleted (Supplemental Figs S5–S7), indicating that photosynthesis was suppressed during the transition to CO_2_ limitation. The addition of CO_2_ (as NaHCO_3_) to the reaction medium restored photosynthetic activity (Fig. 2, Supplemental Figs S5–S7).

During the measurements, the top of the chamber remained open, which enabled O_2_ and CO_2_ to diffuse into or out of the reaction medium. This open system relieved excessive increases in O_2_ in the reaction mixture, which enabled O_2_ to be measured for longer without passing over an undetectable point of the O_2_ electrode. We temporarily closed the chamber to exclude the effects of diffusion of O_2_ for determination of photosynthetic O_2_ evolution rates (Supplemental Figs S5–S7)^13^.

In several earlier studies, it was observed that S. 7942 induced little AEF or NPQ in response to CO_2_ limitation^12^,^23^,^36^. Thus, we used S. 7942 as a control to compare the responses of *E. gracilis* and *P. tricornutum* in this study. In S. 7942, the photosynthetic O_2_ evolution rate decreased in the transition to CO_2_-limited photosynthesis, which was paralleled by the decrease in the relative ETR without NPQ induction (Fig. 2A, Supplemental Fig. S5). The proportional linear relationship between gross photosynthetic activity and relative ETR indicated that S. 7942 hardly drives AEF in the transition to CO_2_ limitation (Supplemental Fig. S5). The increase in NPQ after adding NaHCO_3_ has been observed in a previous study^36^, although the molecular mechanism was unclear.

In both secondary algae, particularly in *E. gracilis*, some relative ETR was uncoupled from the O_2_ evolution rates during CO_2_-limited photosynthesis (Fig. 2B and C, Supplemental Figs S6 and S7), indicating that an AEF partially replaced photosynthesis during CO_2_-limited photosynthesis in these algae. Compared with S. 7942, *E. gracilis* maintained relative ETR uncoupled from the photosynthetic O_2_ evolution rate during CO_2_-limited photosynthesis, which reached approximately half that during CO_2_-saturated photosynthesis (Supplemental Fig. S6). Conversely, NPQ was slightly induced in the transition to CO_2_ limitation in *E. gracilis*, which was not alleviated after at least 5 min after NaHCO_3_ was added (Fig. 2B). These results concurred with those of a previous study^34^.

In the diatom *P. tricornutum*, a dramatic induction of NPQ was observed in the transition to CO_2_ limitation with the suppression of photosynthesis, although the AEF activity was much less, compared with *E. gracilis* (Fig. 2C). The relaxation of NPQ after adding NaHCO_3_ was faster than that in *E. gracilis* (Fig. 2C), which is in agreement with a number of studies of diatomaceous NPQ^24^,^25^,^34^. These data suggest that the NPQ in *E. gracilis* and *P. tricornutum* are derived from different molecular mechanisms.

### Dependences of relative ETR on O_2_ under CO_2_ limitation in S. 7942, *E. gracilis*, and *P. tricornutum*

Diverse responses of photosynthetic electron transport to CO_2_ limitation in S. 7942, *E. gracilis*, and *P. tricornutum* (Fig. 2) suggest different strategies of O_2_ usage when photosynthesis is suppressed. To compare the O_2_ usage of photosynthetic electron transport in these cyanobacterium and algae, we investigated the dependencies of relative ETR on O_2_ during CO_2_-limited photosynthesis. We eliminated O_2_ in the medium by adding glucose, catalase, and glucose oxidase during CO_2_-limited photosynthesis using the method described by Shimakawa *et al*.^23^. We confirmed in advance that the addition of exogenous glucose during illumination did not affect the O_2_ evolution rates and relative ETR in these species (Supplemental Table S1)^23^. The top of the O_2_ electrode chamber was closed to exclude the effects of diffusion of O_2_ and CO_2_ into or out of the reaction medium. It should be noted that there may have been some unintended consequences of using anaerobic conditions. However, removing O_2_ did not affect relative ETR, at least during CO_2_-saturated photosynthesis, in *E. gracilis* or *P. tricornutum* (Supplemental Fig. S8).

**Figure 3:**
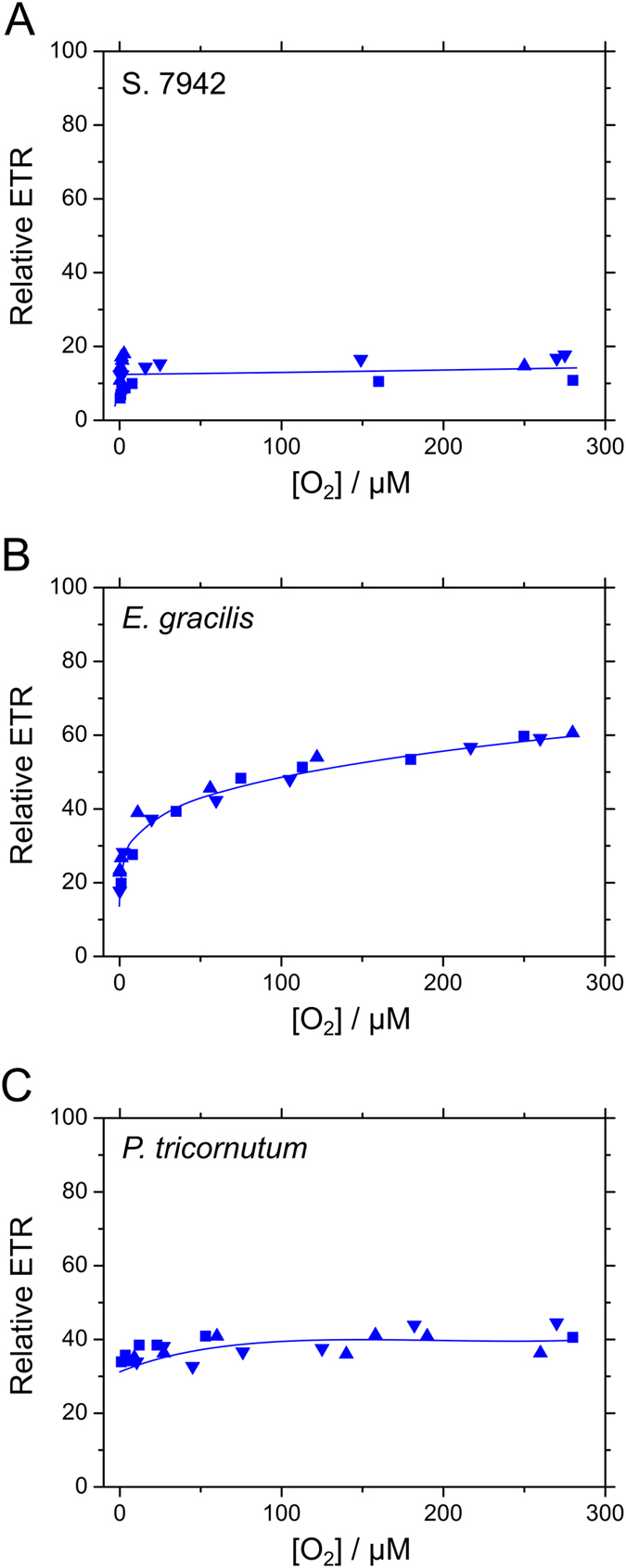
Dependence of relative electron transport rate (ETR) on O_2_ during CO_2_-limited photosynthesis in S. 7942 (**A**), *Euglena gracilis* (**B**), and *Phaeodactylum tricornutum* (**C**). To remove dissolved O_2_, d-glucose (5 mM), catalase (250 units mL^−1^), and glucose oxidase (5 units mL^−1^) was added to the fresh media containing the cells (10 μg Chl *a* mL^−1^). Photon flux densities of white actinic light (AL) were 300 μmol photons m^−2^ s^−1^ for S. 7942 and *E. gracilis*; 400 μmol photons m^−2^ s^−1^ for *P. tricornutum*. Triangles, inverse triangles, and squares represent three independent measurements, respectively.

Compared with S. 7942 and *P. tricornutum*, which required little O_2_ to drive AEF during CO_2_-limited photosynthesis (Fig. 3A and C)^12^,^23^, *E. gracilis* showed a biphasic dependence on O_2_ (Fig. 3B). This indicated that more than two molecular mechanisms functioned as the O_2_-dependent AEF in *E. gracilis*. Conversely, *P. tricornutum* was unlikely to rely on O_2_-dependent AEF to alleviate photo-oxidative damage under CO_2_ limitation, compared with *E. gracilis*. There was residual relative ETR under anaerobic conditions in *E. gracilis* and *P. tricornutum*, which might be derived from an O_2_-insensitive AEF, including CET around PSI and PSII^28^,^29^,^30^,^31^.

## Discussion

**Figure 4:**
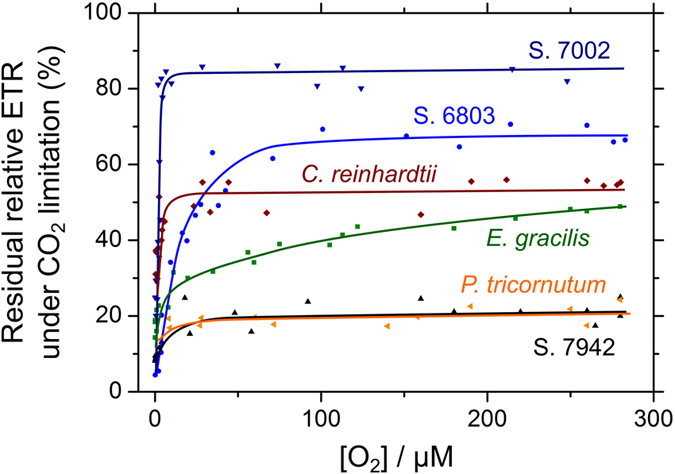
The diversity of O_2_ usage strategies under CO_2_ limitation in cyanobacterial and algal photosynthesis. Shown are cyanobacteria: *Synechocystis* sp. PCC 6803 (S. 6803), *S. elongatus* PCC 7942 (S. 7942), and *Synechococcus* sp. PCC 7002 (S. 7002); the green alga *C. reinhardtii*; the Euglenoid *Euglena gracilis*; and the diatom *Phaeodactylum tricornutum*. Cyanobacterial and algal cells were grown under ambient CO_2_. Residual relative electron transport rate (ETR) under CO_2_ limitation indicates the ratio of relative ETR during CO_2_-limited photosynthesis to that during CO_2_-saturated photosynthesis, which is shown with the dependency on O_2_ in reference to data in this and previous studies^12^,^13^,^22^,^23^.

Figure 4 is a summary diagram of our previous and present results^12^,^13^,^22^,^23^ that presents the diverse O_2_ usage strategies of photosynthetic electron transport to dissipate excess energy under CO_2_ limitation in cyanobacteria, green algae, and two classes of algae with secondary plastids. Oxygenic phototrophs possess a number of molecular mechanisms that protect their cells against photo-oxidative damage by ROS. In this study, we focused on the physiological significance of O_2_ as an alternative ‘safety valve’ in photosynthetic electron transport, and compared responses of photosynthesis to CO_2_ limitation in genetically, phylogenetically, biologically, and ecologically different cyanobacteria and two classes of algae with secondary plastids. These organisms had different pigment compositions (Supplemental Table S2), which made it difficult to quantitatively compare photosynthetic O_2_ evolution rates and relative ETR. Therefore, we simply defined the ratio of relative ETR during CO_2_-limited photosynthesis to that during CO_2_-saturated photosynthesis as residual relative ETR under CO_2_ limitation in each species, and summarized the dependencies on O_2_ as shown in Fig. 4. The cyanobacterium S. 6803, harboring FLV2/4, showed the activation of an O_2_-dependent AEF during CO_2_-limited photosynthesis^13^. This was different from S. 7942, which does not possess FLV2/4 (Fig. 2A)^12^. Conversely, the marine species S. 7002, which does not harbor FLV2/4, maintained a relatively high electron flux to O_2_ during CO_2_-limited photosynthesis owing to the higher contribution of FLV1 and 3 homologs (FLV1/3) to AEF, compared with S. 7942 and S. 6803^22^,^23^. The green alga *Chlamydomonas reinhardtii* drives an O_2_-dependent AEF in the transition from CO_2_-saturated to CO_2_-limited photosynthesis, similar to S. 7002^23^. The dependences of relative ETR on O_2_ under CO_2_ limitation in S. 6803, S. 7942, S. 7002, and *C. reinhardtii* have already been reported in Shimakawa *et al*.^23^. In addition, in *E. gracilis*, the electron flux to O_2_ partially replaced photosynthesis under CO_2_ limitation, while the dependency on O_2_ was different from that in S. 6803, S. 7002, and *C. reinhardtii* (Figs 3B and 4). The biphasic O_2_ dependency of relative ETR in *E. gracilis* indicated that this alga might drive the other AEF, which has low affinity for O_2_ (e.g. photorespiration)^37^,^38^, in addition to the AEF that has high O_2_ affinity. Conversely, the diatom *P. tricornutum* hardly showed O_2_ usage (Figs 3C and 4). Compared with cyanobacteria and algae, C_3_ plants mainly drive photorespiration as an O_2_-dependent AEF that replaces photosynthesis at the CO_2_ compensation point^18^,^19^, whereas this is not observed in the C_4_ plant maize^18^. Overall, there appear to be a number of diverse strategies of O_2_ utilization that prevent photo-oxidative damage under CO_2_ limitation, irrespective of the species of oxygenic phototroph, and O_2_ is essential for some oxygenic phototrophs to protect cells against excess photon energy^21^,^22^.

Overall, dissipating photon energy to O_2_ is not necessarily a universal strategy in oxygenic phototrophs during CO_2_-limited photosynthesis (Fig. 4). In many oxygenic phototrophs, the MAP pathway and respiratory terminal oxidases reduce O_2_ at low concentrations, but in most cases, the rates estimated are less than 10% of CO_2_-saturated gross photosynthesis^39^,^40^,^41^, whereas some species show high activity of MAP pathway *in vivo*^42^. In this study, we measured the activities of the MAP pathway under CO_2_ limitation in S. 7942, *E. gracilis*, and *P. tricornutum* by adding exogenous H_2_O_2_^43^. The maximum activities reached approximately 70%, 25%, and 10% of the gross photosynthetic O_2_-evolution rates in S. 7942, *E. gracilis*, and *P. tricornutum,* respectively (Supplemental Fig. S9). These estimates can be applied to the dependence of relative ETR on O_2_ under CO_2_ limitation in *E. gracilis,* but not in S. 7942 or *P. tricornutum* (Fig. 3). That is, both S. 7942 and *P. tricornutum* probably suppressed the MAP pathway under CO_2_ limitation, and relied upon alternative strategies to dissipate excess photon energy.

There are mechanisms other than O_2_-dependent AEF that function in the protection of cells against photo-oxidative damage, which would explain why there are diverse strategies of O_2_ usage in oxygenic phototrophs. Increased NPQ is broadly used in many oxygenic phototrophs to dissipate excess photon energy, but the molecular mechanisms are unlikely to have the same origin. In the cyanobacterium S. 6803, it is observed that the orange carotenoid protein is expressed and functions in NPQ in response to high light levels^44^. However, in the transition to CO_2_ limitation, no induction of NPQ was observed in S. 7942 or S. 6803 (Fig. 2A)^12^,^23^,^36^. The strategy to enhance NPQ under CO_2_ limitation might not have been widespread in oxygenic phototrophs during their early evolution. The diatom *P. tricornutum* showed a large increase in NPQ under CO_2_ limitation (Fig. 2C), which would be strictly related to ΔpH, some carotenoids, and the gene product of *lhcX*s^24^,^25^,^45^. Conversely, the suppression of electron transport in the cytochrome *b*_6_/*f* complex is stimulated by ΔpH^26^ and reduced the plastoquinone pool^27^, both of which can cause the oxidation of P700 to dissipate excess photon energy^22^. Additionally, O_2_-insensitive AEF, including CET around PSI^28^,^29^ and PSII^30^,^31^ may function to alleviate photo-oxidative damage. Furthermore, phototaxis possibly functions as a main strategy to avoid excess photon energy under CO_2_ limitation in motile algae, such as *E. gracilis*^46^. These O_2_-insensitive strategies to alleviate photo-oxidative damage would enable various oxygenic phototrophs to be independent of O_2_-dependent AEF. Nevertheless, the questions of the benefit (or cost) of O_2_ usage to dissipate excess photon energy remains. There are still many questions over the diverse strategies that oxygenic phototrophs use to counter the detrimental effects of sunlight.

## Methods

### Growth conditions and determination of Chlorophyll

Cyanobacteria and algae were cultured in baffled shake flasks on a rotary shaker (100 rpm) under ambient CO_2_. For all measurements, cells from the exponential growth phase were used.

S. 7942 was cultured in BG-11 medium^47^ under light:dark conditions (25 °C, 16 h, 150 μmol photons m^−2^ s^−1^, fluorescent lamp: 23 °C, 8 h, dark). To quantify Chl, cells were centrifugally harvested and re-suspended by vortexing in 1 mL 100% (v/v) methanol. After subsequent incubation at room temperature for 5 min, the suspension was centrifuged at 10,000 × *g* for 2 min. Total Chl was determined from the supernatant^48^.

*E. gracilis Z* (NIES-48) was photoautotrophically cultured in Cramer-Myers medium^49^ under light:dark conditions (25 °C, 16 h, 150 μmol photons m^−2^ s^−1^, fluorescent lamp: 23 °C, 8 h, dark). Both Chl *a* and Chl *b* were quantified following the above-mentioned method^48^.

*P. tricornutum* (UTEX642) was photoautotrophically cultured in the artificial seawater medium described previously, with the addition of 0.31% half-strength Guillard’s ‘F’ solution^50^,^51^, under light:dark conditions (22 °C, 14 h, 100 μmol photons m^−2^ s^−1^, fluorescent lamp: 20 °C, 10 h, dark). Both Chl *a* and Chl *c* were quantified as described above, except that the cells were re-suspended in a 1 mL mixture (10% [v/v] distilled water, 10% [v/v] dimethyl sulfoxide, and 80% [v/v] acetone)^52^.

### Measurement of O_2_ and Chl fluorescence

Net uptake and evolution of O_2_ was measured simultaneously with Chl fluorescence. Cell samples in freshly prepared media (2 mL, 10 μg Chl *a* mL^−1^) were stirred with a magnetic microstirrer and illuminated with white actinic light (AL) at 25 °C (for S. 7942 and *E. gracilis*) or 20 °C (for *P. tricornutum*). A halogen lamp (Xenophot HLX 64625, Osram, München, Germany) from the LS2 light source (Hansatech, King’s Lynn, UK) was used as the white AL source. O_2_ was monitored continuously using an O_2_ electrode (Hansatech, King’s Lynn, UK) while the measuring cuvette remained open to allow diffusion of O_2_ and CO_2_ between the medium and the air^12^,^13^. The top of the cuvette was temporarily closed (1–3 min) while the O_2_ evolution rate was determined^12^,^13^. Representative raw traces of O_2_ and relative Chl fluorescence in S. 7942, *E. gracilis*, and *P. tricornutum* are shown in Supplemental Figs S5A, S6A and S7A, respectively.

The relative Chl fluorescence originating from Chl *a* was measured using a PAM-Chl fluorometer (PAM-101; Walz, Effeltrich, Germany)^53^,^54^. Pulse-modulated excitation was achieved using an LED lamp with a peak emission at 650 nm. Modulated fluorescence was measured at *λ* > 710 nm (Schott RG9 long-pass filter). The minimum Chl fluorescence (F_o_) was determined from illumination using a measuring light (ML). The steady-state fluorescence (F_s_) was monitored under AL, and 1,000-ms pulses of saturated light (10,000 μmol photons m^−2^ s^−1^) were supplied at arbitrary intervals to determine the maximum variable fluorescence (F_m_′). The fluorescence terminology used in this study follows that of van Kooten and Snel (1990)^55^. The effective quantum yield of PSII, Y(II), was defined as (F_m_′ − F_s_)/F_m_′. Relative ETR at PSII was estimated as the product of Y(II) and photon flux density of white AL. NPQ was calculated as (F_m_ − F_m_′)/F_m_′^56^. For S. 7942, F_m_ was determined in the presence of 3-(3,4-dichlorophenyl)-1,1-dimethylurea to exclude effects of state transition^35^.

To measure the dependence of relative ETR on O_2_ (Fig. 3, Supplemental Fig. S8), we added glucose (5 mM), catalase (250 units mL^−1^, Wako, from bovine liver), and glucose oxidase (5 units mL^−1^, Wako, from *Aspergillus niger*) to the medium with the chamber closed to block the diffusion of air to the medium. After photosynthetic O_2_ evolution rates decreased to 0, we added these agents and evaluated the relative ETR^23^.

Activity of the Mehler-ascorbate peroxidase pathway in cyanobacterial and algal cells was estimated from H_2_O_2_-dependent O_2_ evolution rates during CO_2_-limited photosynthesis^43^. To exclude the effects of catalase, we added hydroxylamine (HA) to the reaction medium at 0.1 mM (for S. 7942 and *P. tricornutum*) or 0.5 mM (for *E. gracilis*).

### Measurement of nitrogen

Cyanobacterial and algal cells were centrifugally harvested and dried overnight at 60 °C. Dried pellets were digested using the Kjeldahl method with sulfuric acid and H_2_O_2_. Total N content was determined using Nessler’s reagent after adding sodium potassium tartrate and NaOH^57^.

## Additional Information

**How to cite this article**: Shimakawa, G. *et al*. Diverse strategies of O_2_ usage for preventing photo-oxidative damage under CO_2_ limitation during algal photosynthesis. *Sci. Rep.* **7**, 41022; doi: 10.1038/srep41022 (2017).

**Publisher's note:** Springer Nature remains neutral with regard to jurisdictional claims in published maps and institutional affiliations.

## Supplementary Material

Supplementary Information

## Figures and Tables

**Figure 1:**
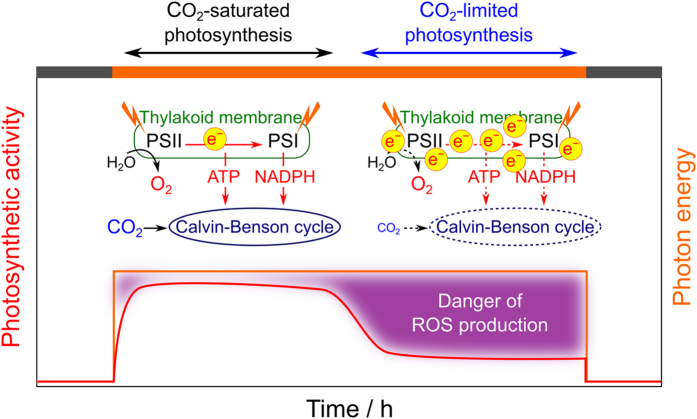
A simple model of photosynthesis. Orange line shows photon energy and red line shows photosynthetic activity. Photosynthesis actively occurs when sufficient CO_2_ is available (CO_2_-saturated photosynthesis, double-headed black arrow). Under CO_2_-limited photosynthesis (double-headed blue arrow), excess photon energy accumulates in a photosynthetic electron transport system located on the thylakoid membranes, which causes the production of reactive oxygen species.
