# Supplementary material for: Diverse strategies of O2 usage for preventing photo-oxidative damage under CO2 limitation during algal photosynthesis
Source: Sci Rep. 2017 Jan 20;7:41022. doi: 10.1038/srep41022 (PMC5247695; doi:10.1038/srep41022)
Supplement: Supplementary Information [file srep41022-s1.pdf]

Diverse strategies of O_2 usage for preventing photo-oxidative damage under CO_2 limitation during algal photosynthesis

Ginga Shimakawa; Yusuke Matsuda; Kensuke Nakajima; Masahiro Tamoi; Shigeru Shigeoka; Chikahiro Miyake

Supplemental Table S1. Effects of D-glucose on photosynthesis in secondary algae

	Control	10 min in D-glucose	Control	10 min in D-glucose
E. gracilis	*O_2 evolution rate / µmol O_2 (mg Chl)$^{-1}$ h^{-1}*		*Relative ETR*	
CO_2 saturation	85 ± 2	86 ± 4	127 ± 3	127 ± 6
CO_2 limitation	8 ± 3	7.1 ± 0.8	69 ± 5	69 ± 4
P. tricornutum	*O_2 evolution rate / µmol O_2 (mg Chl a)$^{-1}$ h^{-1}*		*Relative ETR*	
CO_2 saturation	127 ± 6	130 ± 2	136 ± 7	138 ± 3
CO_2 limitation	3.0 ± 1.5	3.1 ± 1.9	52 ± 3	50 ± 3

Final concentration of D-glucose is 5 mM. Control shows the data before D-glucose is added. Experiments under CO_2 saturation were conducted in the presence of 10 mM $NaHCO_3$. Further, experiments were performed also under CO_2 limitation prepared by the analyses shown in Supplemental Fig. S6 and S7. All of the other experimental procedures are the same as those in Supplemental Fig. S6 and S7. Measurements were independently conducted three times, and the data are shown as means ± SD.

Diverse strategies of O_2 usage for preventing photo-oxidative damage under CO_2 limitation during algal photosynthesis

Ginga Shimakawa; Yusuke Matsuda; Kensuke Nakajima; Masahiro Tamoi; Shigeru Shigeoka; Chikahiro Miyake

Supplemental Table S2. Nitrogen-based Chl contents in cyanobacterial and algal cells used in this study

	Chl content / mg Chl (mg N)$^{-1}$			
	Total Chl	Chl *a*	Chl *b*	Chl $c_{1+}c_2$
**S. 7942**	0.19 ± 0.04	0.19 ± 0.04	-	-
E. gracilis	0.36 ± 0.03	0.32 ± 0.04	0.040 ± 0.014	-
P. tricornutum	0.34 ± 0.04	0.31 ± 0.04	-	0.035 ± 0.003

Measurements were independently conducted three times, and the data are shown as means ± SD

Diverse strategies of O_2 usage for preventing photo-oxidative damage under CO_2 limitation during algal photosynthesis

Ginga Shimakawa; Yusuke Matsuda; Kensuke Nakajima; Masahiro Tamoi; Shigeru Shigeoka; Chikahiro Miyake

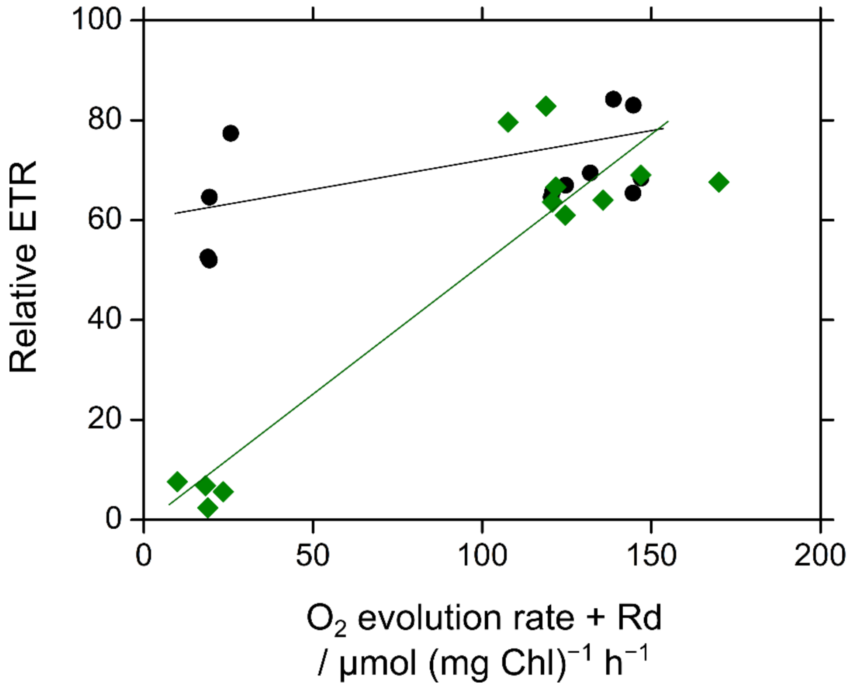

Supplemental Figure S1. The relationship between gross photosynthetic O_2 evolution rate (net O_2 evolution rate + dark respiration rate [Rd]) and relative electron transport rate (ETR) in the cyanobacterium S. 6803 wild type (WT) and the mutant Δ*flv4*. The data plotted were referred from our previous study[13]. Photosynthetic O_2 evolution rates and relative ETR were measured through the transition from CO_2-saturated to CO_2-limited conditions. Black circles, S. 6803 WT; green diamonds, Δ*flv4*. Cells in the reaction mixture (50 mM HEPES-KOH, pH 7.5, 10 μg Chl *a* mL^{-1}) were illuminated with red actinic light (200 μmol photons m^{-2} s^{-1}).

Figure S1. Shimakawa et al.

Diverse strategies of O_2 usage for preventing photo-oxidative damage under CO_2 limitation during algal photosynthesis

Ginga Shimakawa; Yusuke Matsuda; Kensuke Nakajima; Masahiro Tamoi; Shigeru Shigeoka; Chikahiro Miyake

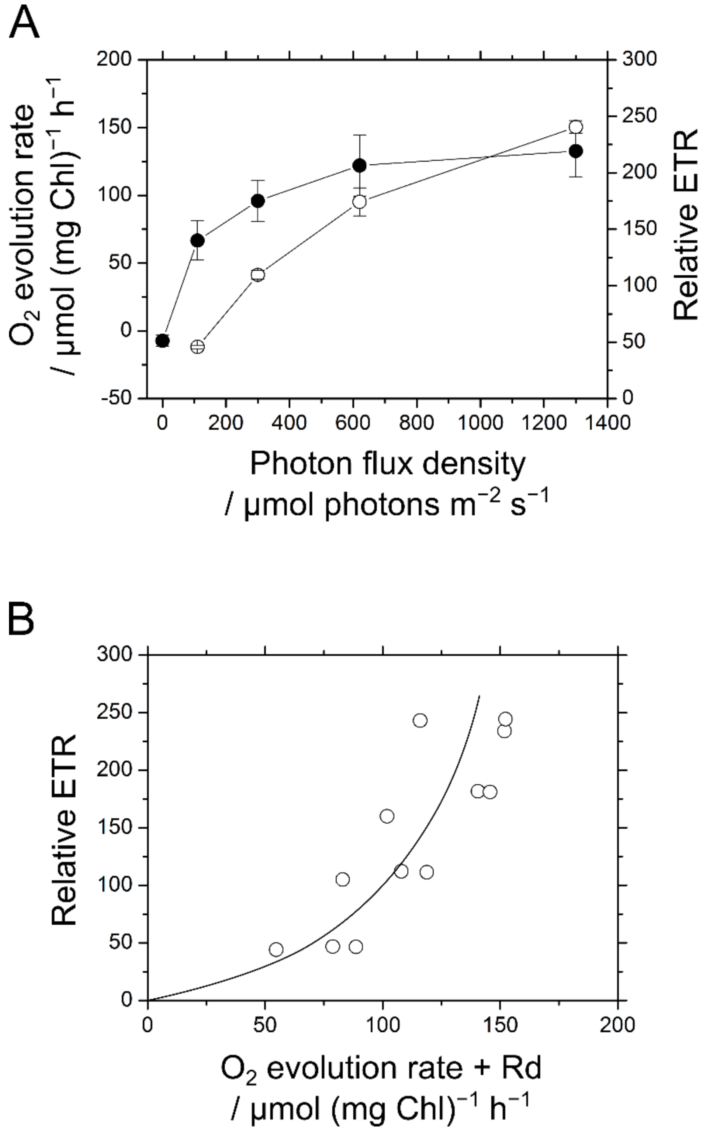

Supplemental Figure S2. Light-response curve of O_2 evolution rate and relative electron transport rate (ETR) during CO_2-saturated photosynthesis in S. 7942. (A) Dependence of O_2 evolution rate (closed circles) and relative ETR (open circles) on photon flux density. Negative values show dark respiration rate (Rd). Reaction media contained cyanobacterial cells (10 μg Chl *a* mL^{-1}) and $NaHCO_3$ (final concentration 10 mM). Measurements were independently taken three times, and the data are shown as means ± SD. (B) The relationship between gross photosynthetic O_2 evolution rate (O_2 evolution rate + Rd) and relative ETR. Data were derived from Supplemental Fig. S2A.

Figure S2. Shimakawa et al.

Diverse strategies of O_2 usage for preventing photo-oxidative damage under CO_2 limitation during algal photosynthesis

Ginga Shimakawa; Yusuke Matsuda; Kensuke Nakajima; Masahiro Tamoi; Shigeru Shigeoka; Chikahiro Miyake

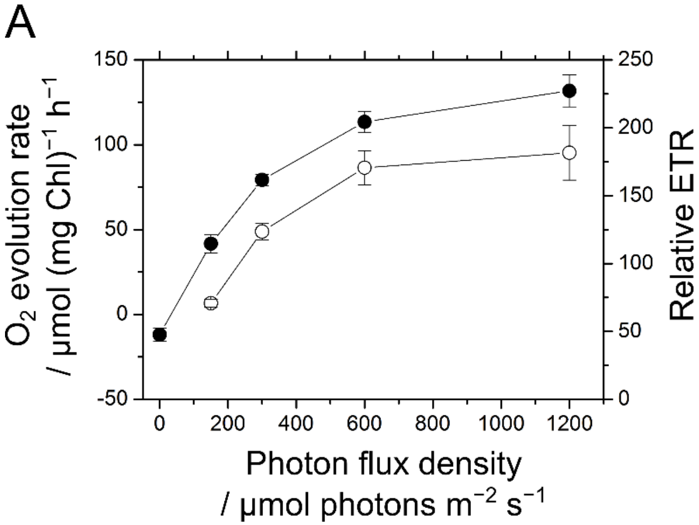

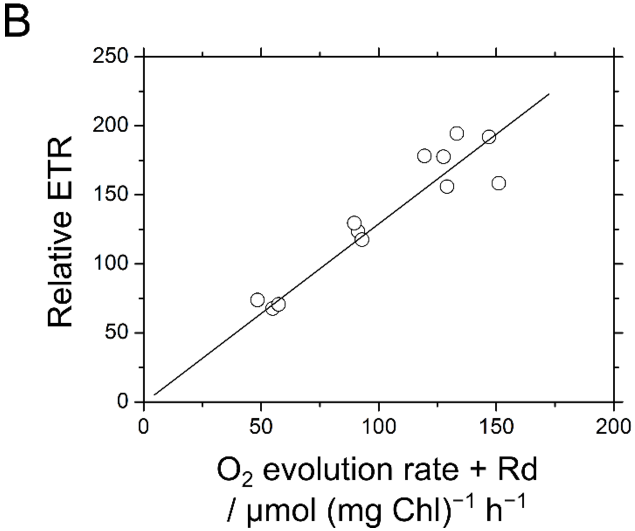

Supplemental Figure S3. Light-response curve of O_2 evolution rate and relative electron transport rate (ETR) during CO_2-saturated photosynthesis in *Euglena gracilis*. (A) Dependence of O_2 evolution rate (closed circles) and relative ETR (open circles) on photon flux density. Negative values show dark respiration rate (Rd). Reaction media contained cyanobacterial cells (10 μg Chl *a* mL^{-1}) and $NaHCO_3$ (final concentration 10 mM). Measurements were independently taken three times, and the data are shown as means ± SD. (B) The relationship between gross photosynthetic O_2 evolution rate (O_2 evolution rate + Rd) and relative ETR. Data were derived from Supplemental Fig. S3A.

Figure S3. Shimakawa et al.

Diverse strategies of O_2 usage for preventing photo-oxidative damage under CO_2 limitation during algal photosynthesis

Ginga Shimakawa; Yusuke Matsuda; Kensuke Nakajima; Masahiro Tamoi; Shigeru Shigeoka; Chikahiro Miyake

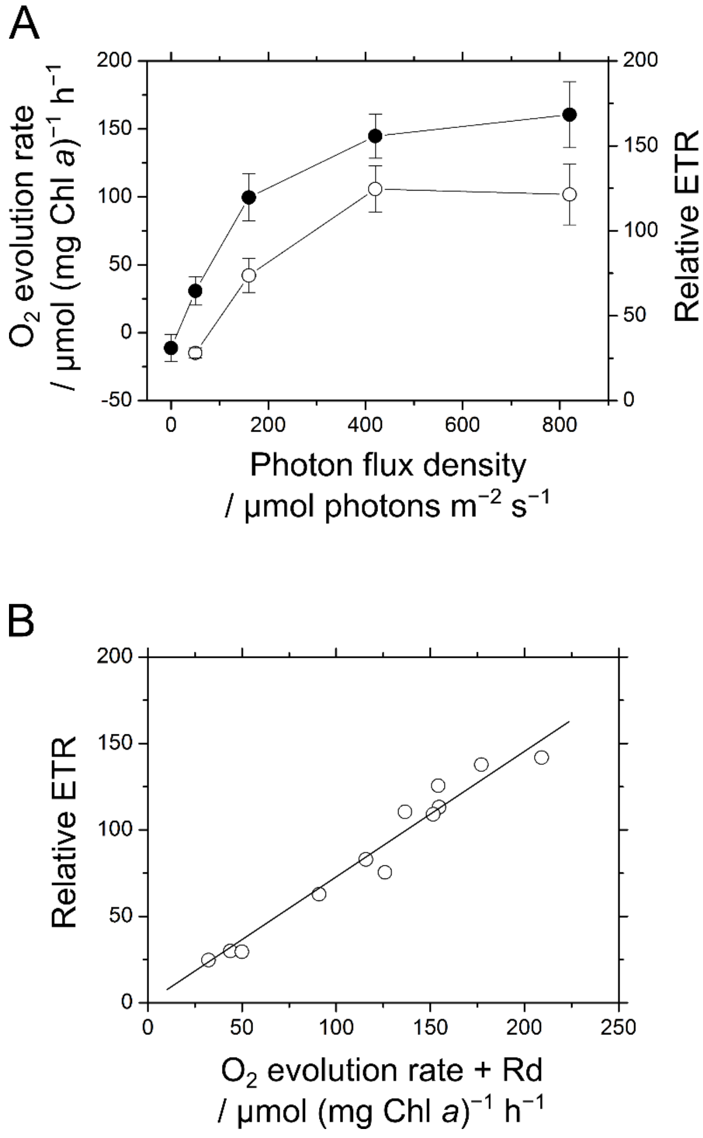

Supplemental Figure S4. Light-response curve of O_2 evolution rate and relative electron transport rate (ETR) during CO_2-saturated photosynthesis in *Phaeodactylum tricornutum*. (A) Dependence of O_2 evolution rate (closed circles) and relative ETR (open circles) on photon flux density. Negative values show dark respiration rate (Rd). Reaction media contained cyanobacterial cells (10 µg Chl *a* mL^{-1}) and $NaHCO_3$ (final concentration 10 mM). Measurements were independently taken three times, and the data are shown as means ± SD. (B) The relationship between gross photosynthetic O_2 evolution rate (O_2 evolution rate + Rd) and relative ETR. Data were derived from Supplemental Fig. S4A.

Figure S4. Shimakawa et al.

Diverse strategies of O_2 usage for preventing photo-oxidative damage under CO_2 limitation during algal photosynthesis

Ginga Shimakawa; Yusuke Matsuda; Kensuke Nakajima; Masahiro Tamoi; Shigeru Shigeoka; Chikahiro Miyake

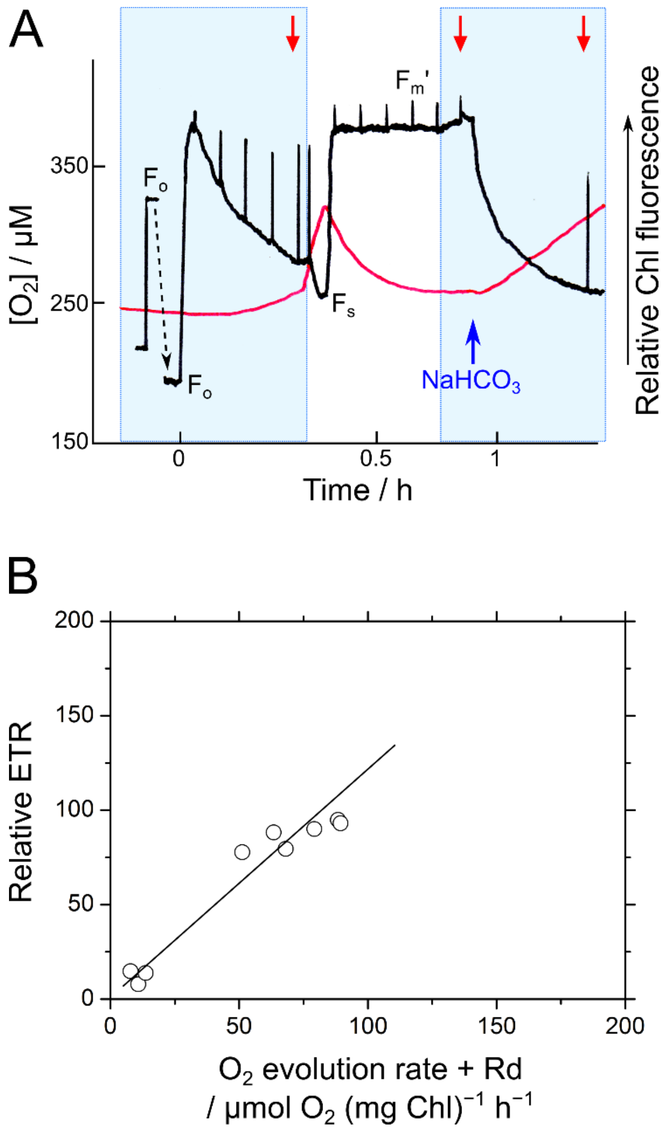

Supplemental Figure S5. Response of photosynthesis to CO_2 limitation in S. 7942. (A) Raw trace showing the time course of O_2 in the reaction media (red line) and relative Chlorophyll (Chl) fluorescence of the cells (black line). Illumination with white actinic light (AL) (300 μmol photons m^{-2} s^{-1}) began at 0. Chl fluorescence parameters had the usual definitions (F_o, minimum fluorescence determined under ML; F_s, steady-state fluorescence under AL; F_m', maximum variable fluorescence under saturating light). Reaction media contained cyanobacterial cells (10 μg Chl *a* mL^{-1}). $NaHCO_3$ (final concentration 10 mM) was added at the point indicated by blue arrow. Blue shading indicates that the top of the O_2 electrode chamber was closed and that the measurement time scales (*x*-axis) were reduced to 1/10. O_2 evolution rates were determined at the times indicated by red arrows. Measurements were taken three times and representative data are shown. (B) The relationship between gross photosynthetic O_2 evolution rate (net O_2 evolution rate + dark respiration rate [Rd]) and relative electron transport rate in the three independent measurements.

Figure S5. Shimakawa et al.

Diverse strategies of O_2 usage for preventing photo-oxidative damage under CO_2 limitation during algal photosynthesis

Ginga Shimakawa; Yusuke Matsuda; Kensuke Nakajima; Masahiro Tamoi; Shigeru Shigeoka; Chikahiro Miyake

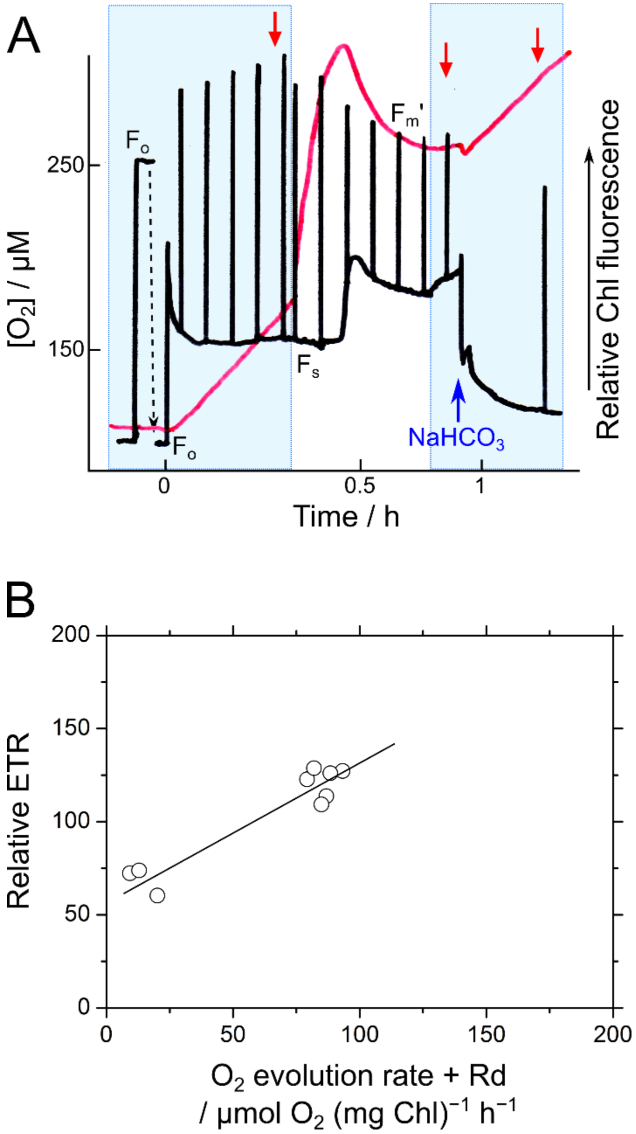

Supplemental Figure S6. Response of photosynthesis to CO_2 limitation in *Euglena gracilis*. (A) Raw trace showing the time course of O_2 in the reaction media (red line) and relative Chlorophyll (Chl) fluorescence of the cells (black line). Illumination with white actinic light (AL) (300 µmol photons m^{-2} s^{-1}) began at 0. Chl fluorescence parameters had the same definitions as described previously (see “Supplemental Figure S5”). Reaction media contained algal cells (10 µg Chl *a* mL^{-1}). $NaHCO_3$ (final concentration 10 mM) was added at the point indicated by blue arrow. Blue shading indicates that the top of the O_2 electrode chamber was closed and that the measurement time scales (*x*-axis) were reduced to 1/10. O_2 evolution rates were determined at the times indicated by red arrows. Measurements were taken three times and representative data are shown. (B) The relationship between gross photosynthetic O_2 evolution rate (net O_2 evolution rate + dark respiration rate [Rd]) and relative electron transport rate in the three independent measurements.

Figure S6. Shimakawa et al.

Diverse strategies of O_2 usage for preventing photo-oxidative damage under CO_2 limitation during algal photosynthesis

Ginga Shimakawa; Yusuke Matsuda; Kensuke Nakajima; Masahiro Tamoi; Shigeru Shigeoka; Chikahiro Miyake

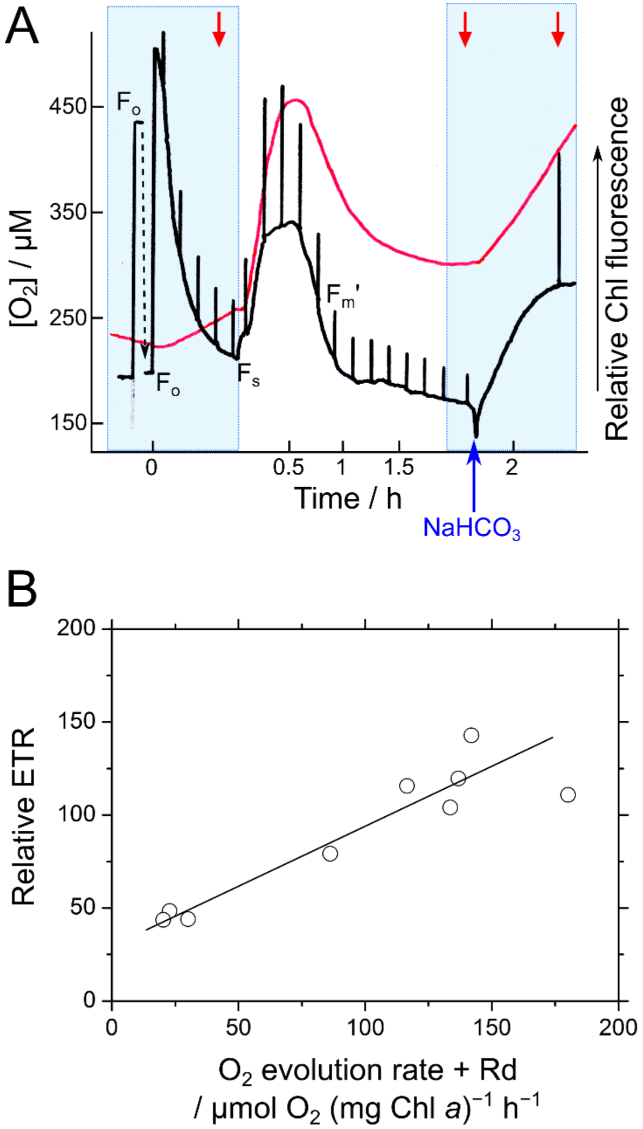

Supplemental Figure S7. Response of photosynthesis to CO_2 limitation in *Phaeodactylum tricornutum*. (A) Raw trace showing the time course of O_2 in the reaction media (red line) and relative Chlorophyll (Chl) fluorescence of the cells (black line). Illumination with white actinic light (AL) (300 µmol photons m^{-2} s^{-1}) began at 0. Chl fluorescence parameters had the same definitions as described previously (see "Supplemental Figure S5"). Reaction media contained algal cells (10 µg Chl *a* mL^{-1}). $NaHCO_3$ (final concentration 10 mM) was added at the point indicated by blue arrow. Blue shading indicates that the top of the O_2 electrode chamber was closed and that the measurement time scales (*x*-axis) were reduced to 1/10. O_2 evolution rates were determined at the times indicated by red arrows. Measurements were taken three times and representative data are shown. (B) The relationship between gross photosynthetic O_2 evolution rate (net O_2 evolution rate + dark respiration rate [Rd]) and relative electron transport rate in the three independent measurements.

Figure S7. Shimakawa et al.

Diverse strategies of O_2 usage for preventing photo-oxidative damage under CO_2 limitation during algal photosynthesis

Ginga Shimakawa; Yusuke Matsuda; Kensuke Nakajima; Masahiro Tamoi; Shigeru Shigeoka; Chikahiro Miyake

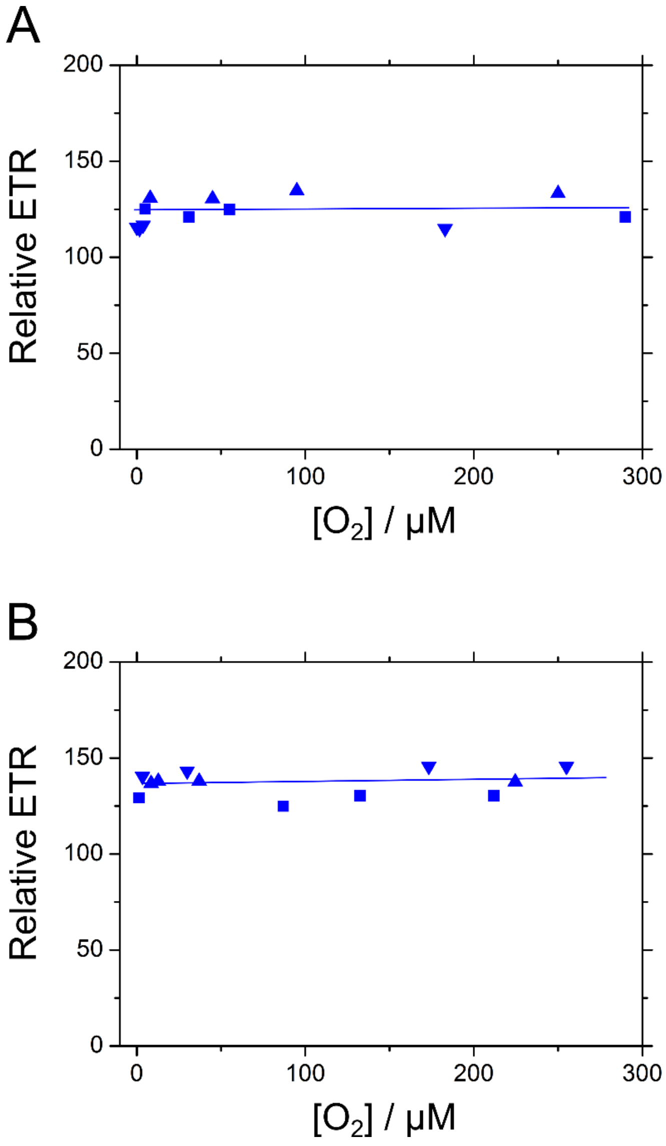

Supplemental Figure S8. Dependence of relative electron transport rate (ETR) on O_2 during CO_2-saturated photosynthesis in *Euglena gracilis* (A) and *Phaeodactylum tricornutum* (B). To remove dissolved O_2, D-glucose (5 mM), catalase (250 units mL^{-1}), and glucose oxidase (5 units mL^{-1}) was added to the fresh media containing the cells (10 μg Chl *a* mL^{-1}). Photon flux densities of white actinic light (AL) were 300 μmol photons m^{-2} s^{-1} for *E. gracilis* and 400 μmol photons m^{-2} s^{-1} for *P. tricornutum*. CO_2-saturated conditions were prepared in the presence of 10 mM $NaHCO_3$. Triangles, inverse triangles, and squares represent three independent measurements, respectively.

Figure S8. Shimakawa et al.

Diverse strategies of O_2 usage for preventing photo-oxidative damage under CO_2 limitation during algal photosynthesis

Ginga Shimakawa; Yusuke Matsuda; Kensuke Nakajima; Masahiro Tamoi; Shigeru Shigeoka; Chikahiro Miyake

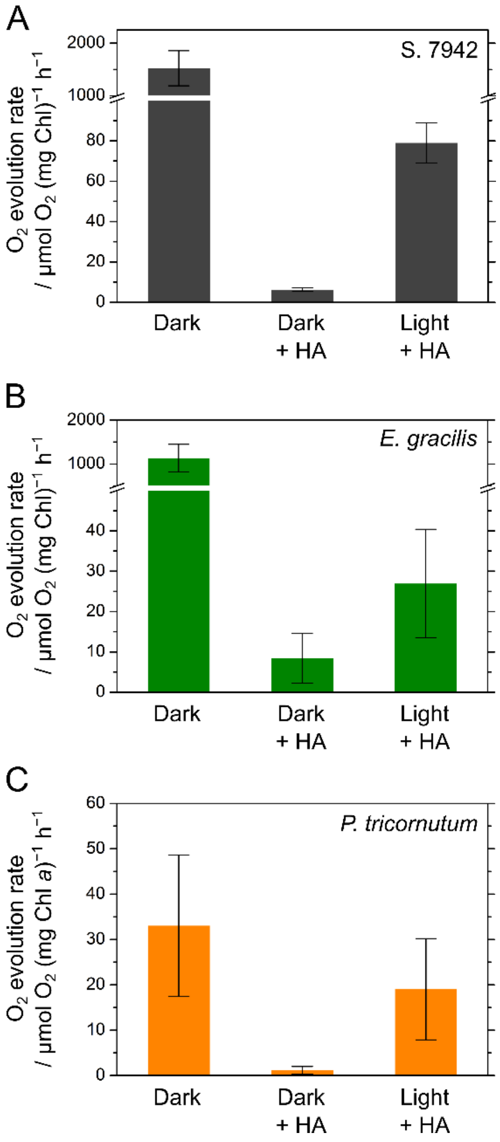

Supplemental Figure S9. H_2O_2-induced O_2 evolution in S. 7942, *Euglena gracilis*, and *Phaeodactylum tricornutum*. H_2O_2 (0.5 mM) was added to the reaction media containing cyanobacterial or algal cells (10 µg Chl *a* mL^{-1}) in the dark and during CO_2-limited photosynthesis. Photon flux densities of white actinic light (AL) were 300 µmol photons m^{-2} s^{-1} for *E. gracilis* and 400 µmol photons m^{-2} s^{-1} for *P. tricornutum*. To exclude O_2 evolution by catalase reaction, we added hydroxylamine (HA) at 0.1 mM for S. 7942 and *P. tricornutum*, and 0.5 mM for *E. gracilis*. For the measurements during CO_2-limited photosynthesis, we added H_2O_2 after photosynthetic O_2 evolution rate reduced to almost 0, with the top of the O_2 electrode chamber closed. Measurements were independently taken three times, and the data are shown as means ± SD.

Figure S9. Shimakawa et al.
